# Removal of Methyl Red from Aqueous Solution Using Polyethyleneimine Crosslinked Alginate Beads with Waste Foundry Dust as a Magnetic Material

**DOI:** 10.3390/ijerph19159030

**Published:** 2022-07-25

**Authors:** Hyunsoo Kim, Oyunbileg Purev, Eunji Myung, Nagchoul Choi, Kanghee Cho

**Affiliations:** 1 Department of Energy and Resource Engineering, Chosun University, Gwangju 61452, Korea; star8538@naver.com (H.K.); oyunbileg@chosun.kr (O.P.); 2 Green-Bio Research Facility Center, Seoul National University, Pyeongchang-gun 25354, Korea; ej6865@snu.ac.kr; 3 Research Institute of Agriculture and Life Sciences, Seoul National University, Seoul 08826, Korea; nagchoul@snu.ac.kr

**Keywords:** waste foundry dust, sodium alginate, polyethylenimine, methyl red removal, adsorption

## Abstract

In this study, a cost-effective adsorbent based on sodium alginate (SA) with waste foundry dust (WFD) was fabricated for the removal of methyl red (MR) from aqueous media. However, the utilization of WFD/SA beads to remove anionic dyes (such as MR) from effluents has limitations associated with their functional groups. To improve the adsorption performance, WFD/SA-polyethyleneimine (PEI) beads were formed via PEI crosslinking onto WFD/SA beads, which could be attributed to the formation of amide bonds from the carboxyl and amino groups due to the change of N-H bonds in the reaction. The Fourier transform infrared (FTIR) and X-ray photoelectron spectroscopy (XPS) results indicated that PEI was crosslinked on the WFD/SA via a chemical reaction. In the FTIR spectra of WFD/SA-PEI, peaks of the –COO (asymmetric) stretching vibration shifted to 1598 and 1395 cm^−1^, which could be attributed to the hydrogen-bonding effect of the N–H groups in PEI. In the N1s spectrum, three deconvoluted peaks were assigned to N in –N= (398.2 eV), –NH/–NH_2_ (399.6 eV), and NO_2_ (405.2 eV). WFD/SA-PEI beads were assessed and optimized for aqueous MR adsorption. The WFD/SA-PEI beads showed a high removal efficiency for MR (89.1%) at an initial concentration of 1000 mg/L, and presented a maximum MR adsorption capacity of 672.7 mg/g MR. The adsorption process showed a good fit with the pseudo-second-order kinetic model and the Langmuir adsorption isotherm model. The amino and hydroxyl groups in the WFD/SA-PEI beads facilitate strong hydrogen bonding and electrostatic interactions. Moreover, these WFD/SA-PEI beads were easily recovered after the adsorption process.

## 1. Introduction

Metal casting is one of the basic processes in the manufacturing industry. It is an important means of providing blanks for mechanical parts and is an integral part of many industrial processes [1]. The foundry industry is recognized as a potential source of environmental pollution [2]. Presently, substantial amounts of slag and other waste are generated from steel manufacturing processes. Most of the produced by-products, such as foundry sand, are initially stockpiled in the foundries and recycled several times in various foundry operations and treated for application in cement mixtures and recyclable materials [3]. The main compounds in waste foundry sand (WFS), including quartz and aluminum oxides, can form complex aluminate silicates at high temperatures [4]. Therefore, it is generally believed that foundry sand has good mechanical properties, and its utilization in construction materials has been reported [5,6].

The foundry sand is produced by ferrous foundries (ductile iron, grey iron, and steel), and foundry dust is the fine dust produced during the production of castings from blast furnaces [6]. Foundry dust is considered as the ultimate waste, and the solid materials collected from the gas treatment units before the gases are released into the atmosphere account for the second largest proportion of total waste during the casting process. The common constituent in waste foundry dust (WFD) produced by foundries is iron-containing minerals, primarily consisting of Fe and O in the form of magnetite (Fe_3_O_4_), owing to the processes depending on reduction/oxidation conditions and rapid cooling after combustion processes [7]. In addition, in the process of capturing gases, CaO is used to neutralize the acid gas generated under elevated temperatures during the production of casting [8,9]. Despite its potential utility as a material, WFD is disposed of in industrial landfills, and relatively little research has been conducted on the resource utilization of foundry dust.

Previous studies have reported the use of WFD for heavy metal removal. For example, Rha and Jo (2021) used WFD as an adsorbent to adsorb As^3+^ and Cr^6+^ from aqueous solutions [10]. It was also found that WFD can be used as a reactive material, which causes adsorption, precipitation, and redox reactions by the oxidation of Fe(II). Iron oxide materials, such as magnetite, have been used as supports because of their physicochemical stability, magnetic properties, and ease of modification with organic groups. WFD-based geopolymers have been successfully applied as adsorbents for the removal of Pb^2+^ and Ni^2+^ from aqueous solutions [11].

Dyes are released into water mainly from the textile, leather, and synthetic color production industries [12]. The presence of synthetic dyes in effluents has been implicated in water pollution. This may lead to the incomplete adhesion of the dyes to the substrates during coloring. Owing to this toxic potential, the presence of dyes as pollutants in wastewater can cause significant health issues if they are not adequately treated, leading to severe diseases and disorders. Although several methods of dye removal have been reported, adsorption has been found to be the most effective method, with promising results, including low operational costs, a high treatment speed, and operational stability [13,14].

Recently, researchers have enhanced the dye removal capacity of adsorbents using waste materials of agricultural origin because of their environmental friendliness and low cost [15]. In previous studies, the use of agricultural waste materials through physical and chemically modified plant-based adsorbents has received considerable attention [16]. They used composite materials, such as metal oxides/hydroxides, magnetic materials, LDHs, and polymers [17,18]. In this regard, magnetic materials are widely used as supports for the synthesis of functionalized adsorbents owing to their low toxicity and ease of synthesis [19,20,21]. However, magnetic materials are susceptible to acidic conditions, including sensitivity to oxidation, which might decrease their dispersion ability and reactivity [22,23]. Notably, the most effective method of utilizing magnetic materials is to encapsulate magnetic materials in a suitable matrix. Therefore, encapsulation is an effective approach for the protection of magnetic materials. In this regard, several biopolymer materials have been reported, such as chitosan, pectin, and cellulose, which have a larger specific surface area, functional groups, and adjustable surface chemistry. Among these materials, sodium alginate (SA) consists of M block (D-mannuronic acid) and G block (L-guluronic acid) units, and has been used extensively for inorganic material encapsulation owing to its abundant hydroxyl and carboxyl groups [24,25].

Over the past few decades, many studies have shown that various adsorptive materials, including SA, possess a good adsorption potential for dye removal from aqueous environments. Unfortunately, synthetic dyes contain diverse chemicals according to their dissociation behavior in aqueous solutions. For instance, their dye characteristics include acid when negatively charged, basic when positively charged, and reactive when anionic [26]. Therefore, a surface-modification technique was used to overcome these limitations. The addition of functional groups has been reported to enhance the characteristics of low-cost adsorbents, such as waste materials. Polyethylenimine (PEI) is a non-toxic polymer that efficiently removes anionic dyes and plays a favorable role in modifying the substrate surface [27,28]. PEI also has a cationic polyamine containing a large number of amine groups on macromolecules and is protonated under acidic conditions [29]. It has a high content of amino and hydroxyl functional groups, which are immobilized with other materials to be applied in the adsorption of dyes [30,31,32]. Effective as they are, the aforementioned modified adsorbents still bear drawbacks, such as the difficulty in the separation problem in the solution.

Therefore, to improve the disadvantage of difficulty in separation and improve the adsorption capacity, we fabricated a magnetic adsorbent for the removal of methyl red (MR) from an aqueous solution, in which SA was used as the basic material. In general, it has been previously reported that Ca^2+^ has been used as an effective crosslinker in the preparation of SA hydrogel [33]. However, this method has a single-network structure, which indicates that the prepared calcium alginate composite beads have poor selectivity and stability. Accordingly, a method of chemically modifying SA with PEI comprising abundant amino groups was employed in this study. Reactive dyes are azo-based chromophores combined with different types of reactive groups; compounds found in colored wastewater show low biodegradability and are stable in the presence of oxidizing agents. The MR was selected based on the characteristic properties of reactive dyes, given their dissociation behavior in aqueous solutions. The powdered WFD has limitations as an adsorbent. Therefore, there is scope for improving the WFD. In this study, a magnetic adsorbent was developed by encapsulating WFD as a recycling material acting as Fe_3_O_4_ magnetic in calcium–alginate beads. The WFD/SA beads were then modified with PEI to introduce active amino sites onto the surface. The obtained composite beads were characterized in detail to analyze their components, and the MR removal efficiency from an aqueous solution was investigated.

## 2. Materials and Methods

### 2.1. Chemicals and Reagents

WFD was obtained from a foundry plant in Incheon, South Korea, and was used as the magnetic material in this study. Sodium alginate (SA, 90%) and calcium chloride (CaCl_2_, ≥98%) were purchased from Duksan Pure Chemicals (Ansan-si, Korea), MR (EP grade, C_15_H_15_N_3_O_2_) from Samchun Chemicals (Seoul, Korea), Branched polyethylenimine (PEI, 50% *w*/*w*) from Sigma-Aldrich Inc. (St. Louis, MO, USA) and glutaraldehyde (GA, EP grade, 25% solution in water). The properties of MR (including color, dissociation constant, molecular weight, maximum adsorption wavelength, and structural formula) are presented in Table 1.

### 2.2. Preparation of WFD/SA-PEI

**Figure 1:**
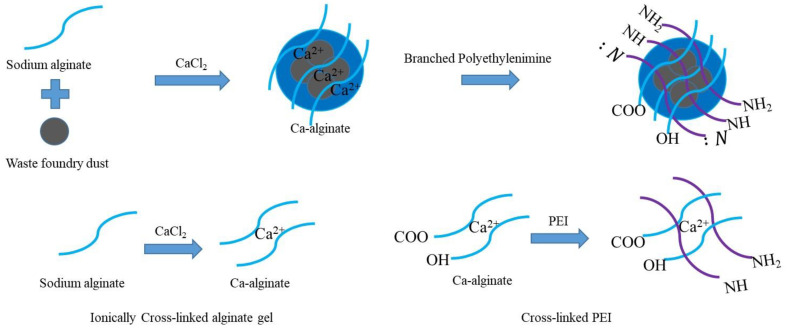
Schematic diagram for the preparation of WFD/SA-PEI.

Prior to the preparation of PEI-functionalized SA, magnetic SA was prepared by modifying the approach by Fan et al. (2019) [34]. Our objective was to obtain magnetic adsorbents with WFD to achieve better separation after use. Briefly, WFD (2% *w*/*v*) was dispersed in deionized water and stirred for 30 min for even mixing. A 2% (*w*/*v*) SA solution was prepared by mixing 2 g of SA in 100 mL of deionized water with stirring for 3 h. The WFD solution was then slowly added to the mixture to ensure that the SA was fully mixed. When the mixture became homogeneous, it was dropped into a stirred solution containing 4% (*w*/*v*) CaCl_2_ using a peristaltic pump at a speed of 0.2 mL/min. This leads to the formation of a calcium alginate shell via ionic crosslinking. Subsequently, the WFD/SA beads were mixed in 100 mL of 1.5% PEI solution and stirred for 24 h to allow them to react with PEI. The wet beads were transferred into 100 mL of 2% (*w*/*v*) glutaraldehyde solution for the crosslinking reaction. The related preparation process is illustrated in Figure 1.

### 2.3. Batch Experiments

Batch experiments were conducted for MR removal using WFD/SA-PEI beads. Adsorption experiments were performed with a 50 mL polypropylene conical tube containing 30 mL solution of the WFD/SA-PEI beads (adsorbent dose = 1–10 g/L) and MR (1000 mg/L) at pH 4.0, unless stated otherwise. The samples were shaken in a shaking incubator at 120 rpm for 24 h. Duplicate tests were run to ensure data quality, and the mean values were calculated with standard deviations of less than 5%.

Kinetic experiments (adsorbent dose = 1, 5, and 10 g/L; initial MR concentration = 1000 mg/L) were performed at reaction times ranging from 10 min to 24 h. Equilibrium isotherm experiments (adsorbent dose = 1, 5, and 10 g/L) were conducted at initial MR concentrations ranging from 100 to 1000 mg/L. After adsorption was complete, the remaining MR in the solution was determined by measuring its absorbance at 520 nm. The effects of the initial solution pH on the MR removal were evaluated at pH values ranging from 1 to 9. The solution pH was adjusted using 0.1 M NaOH and 0.1 M HCl solutions, and pH was measured using a pH probe (9107BN, Thermo Fisher Scientific, Waltham, MA, USA). The effect of co-existing anions, including chloride, nitrate, and sulfate, on MR removal was investigated using various initial co-existing ion concentrations.

The MR removal capacity (*q_e_*, mg/g) can be calculated with the following equation:

(1) $$q_{e}=\frac{C_{i}-C_{f}}{C_{a}}$$

where *C_i_* is the MR concentration in the aqueous phase before the reaction (mg/L), and *C_f_* is the MR concentration in the aqueous phase after the reaction (mg/L), and *C_a_* is the dose of the WDS (g/L).

Kinetic adsorption data were analyzed using the following nonlinear forms of the pseudo-first-order model (Equation (2)) and pseudo-second order (Equation (3)) models:

(2) $$q_{t}=q_{e}\left[1-\exp(-k_{1}t\right]$$

(3) $$q_{t}=\frac{k_{2}q_{e}^{2}t}{1+k_{2}q_{e}t}$$

where *q_t_* is the amount of adsorbed MR per unit mass of absorbent at time *t* (mg/g) and q_e_ is the amount of adsorbed MR per unit mass of absorbent at equilibrium (mg/g). *k*_1_ is the pseudo-first-order rate constant (1/min) and *k*_2_ is the pseudo-second-order rate constant (g/mg/min).

The equilibrium sorption data were analyzed using the following nonlinear forms of Langmuir (Equation (4)) and Freundlich (Equation (5)) isotherm models:

(4) $$q_{e}=\frac{Q_{m}K_{L}C_{e}}{1+K_{L}C_{e}}$$

(5) $$q_{e}=K_{F}C_{e}^{1/n}$$

where *Q_m_* is the maximum mass of adsorbed MR per unit mass of adsorbent (adsorption capacity, mg/g) and *C_e_* is the concentration of MR in the aqueous solution at equilibrium (mg/L). *K_L_* is the Langmuir constant related to the binding energy (L/g), *K_F_* is the Freundlich distribution coefficient (L/mg), and 1/*n* is the Freundlich constant.

### 2.4. Data Analysis

The following equations for the determination of coefficient (*R*^2^), chi-square coefficient (*χ*^2^), and sum of the squared error (SSE) were used to analyze the adsorption data and confirm their fit to the model:

(6) $$R^{2}=\frac{\sum_{i=1}^{m}{\left(y_{c}-\bar{y_{e}}\right)}_{i}^{2}}{\sum_{i=1}^{m}{\left(y_{c}-\bar{y_{e}}\right)}_{i}^{2}+\sum_{i=1}^{m}{\left(y_{c}-y_{e}\right)}_{i}^{2}}$$

(7) $$\chi^{2}=\overset{m}{\underset{i=1}{\sum }}{\left[\frac{{\left(y_{e}-y_{c}\right)}^{2}}{y_{c}}\right]}_{i}$$

(8) $$\mathrm{SSE}=\overset{n}{\underset{i=1}{\sum }}{\left(y_{e}-y_{c}\right)}^{2}$$

where $y_{c}$ is the removal capacity calculated from the model, $y_{e}$ is the removal capacity measured from the experiment and $\bar{y_{e}}$ is the average measured removal capacity.

### 2.5. Analytical Methods

The WFD was analyzed via X-ray diffraction (XRD) (X’Pert Pro MRD, PANalytical, Almelo, The Netherlands). CuKα X-rays were used at an acceleration voltage of 40 kV and a current of 30 mA. The sample was analyzed at 2θ values of 10–70° to determine the mineral phase composition. The elemental composition of the WFD was determined by X-ray fluorescence (XRF) spectrometry (S4 PIONEER, Bruker AXS, Karlsruhe, Germany). The average particle size of the WFD was determined using a particle size analyzer (Mastersizer 2000, Malvern Panalytical Ltd., Malvern, UK).

To determine the potential release of toxic elements from the samples, the Korean standard leaching test (KSLT) was conducted using a modified method. The modified KSLT was performed to analyze the release characteristics of toxic elements in the samples at various pH values. Briefly, 5 g of the sample was added to 50 mL of water at various initial pH values (1–9) and agitated at 200 rpm for 24 h. After the modified KSLT, the effluent was collected and filtered through a 0.45 μm membrane filter. The effluent concentration in the filtrate was measured using ICP-OES (Perkin Elmer Optima Model 5300DV, Waltham, MA, USA).

The magnetic properties of the WFD/SA beads were observed using a vibrating sample magnetometer at room temperature (VSM; LakeShore 7407-S, Lake Shore Cryotronics, Inc., Westerville, OH, USA). The morphology and surface structure of the WFD/SA-PEI beads were analyzed using field-emission scanning electron microscopy (FE-SEM, S4800, Hitachi, Tokyo, Japan) with EDS (ISIS310, Jeol, Tokyo, Japan). A Fourier-transform infrared (FTIR) spectroscopy (Nicolet 6700, Thermo Fisher Scientific) was used to obtain the infrared spectra before and after the sorption experiments. X-ray photoelectron spectroscopy (XPS; Sigma Probe, Kratos Analytical, Shimadzu, Kyoto, Japan) with Al Kα radiation (hv = 1253.6 eV) was employed to analyze the chemical bonding and elements before the sorption experiments. The zeta potential of the WFD/SA-PEI beads under different pH conditions was characterized by zeta potential measurements performed using a Zetasizer Nano Analyzer (ZS 90, Malvern, Worcestershire, UK). N_2_ adsorption–desorption isotherm analysis was performed using a surface area analyzer (BELSORP-max, BEL Japan Inc., Tokyo, Japan).

**Figure 2:**
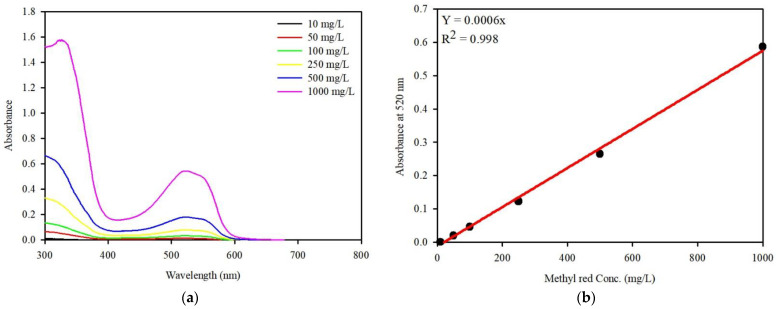
UV-Vis spectrum of MR at (**a**) various concentrations and (**b**) calibration curve for MR.

UV/visible spectroscopic measurements (Aquamate Plus, Thermo Fisher Scientific) were conducted to determine the MR concentration. A calibration curve was obtained with a series of standard MR concentrations ranging from 10 to 1000 mg/L using the absorbance of MR at 520 nm (Figure 2). The coexisting anion concentration in the filtrate was measured using IC (883 Basic IC Plus, Metrohm, AG, Switzerland).

## 3. Results and Discussion

### 3.1. Characterization of WFD

**Figure 3:**
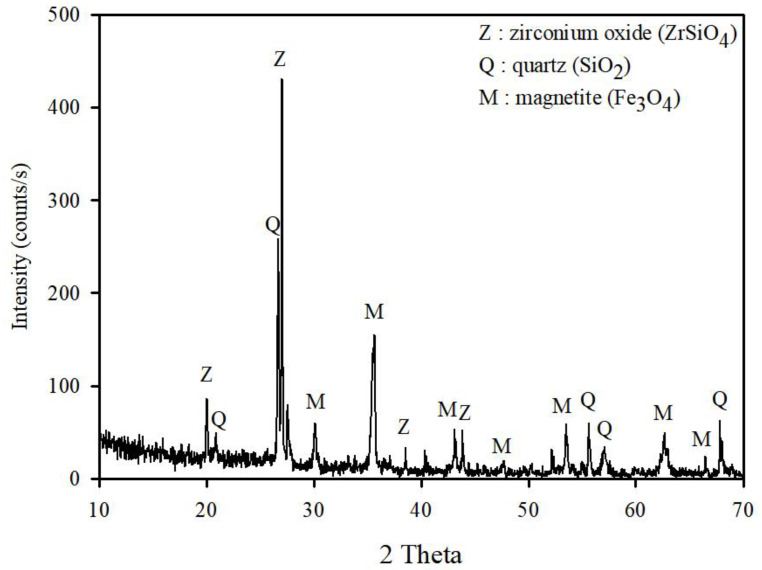
X-ray diffraction patterns of WFD.

The WFD was used for XRD and XRF analyses for mineralogical and chemical determination, respectively. The mineral composition of the WFD was determined via XRD, which revealed that the sample consisted of magnetite, zirconium oxide, and quartz (Figure 3). The Fe, Si, and Zr contents were as high as 27.9%, 11.9%, and 7.47%, respectively. The major element compositions and mineralogical characteristics of the samples are listed in Table 2. The average particle size of IFA was determined using a particle-size analyzer. The median particle size (D50) of IFA was 24.9 µm.

The modified KSLT was conducted as a function of the initial solution pH to examine the leaching of toxic elements from the WFD (Table 3). Generally, the leachate pH significantly influences the leaching behavior of toxic elements such as heavy metals to a greater extent under lower pH values. The heavy metal with the highest concentration in the WFD was Ca, followed by Cu and Fe at pH < 3, with toxic elements such as As, Cd, and Cr having detection limits (<0.1 mg/L) for all pH values used in this study. For the WFD, the leaching concentrations of Cu and Fe decreased as a function of the initial solution pH, which increased from 1 to 9 owing to the dissolution of Ca in the leachate. These results indicate that WFD has a low possibility of secondary contamination owing to the lower dissolution rates of WFD minerals. Consequently, WFD can be used as a magnetic material for the characterization of alginate.

### 3.2. Characterization of WFD/SA Bead and WFD/SA-PEI Bead

**Figure 4:**
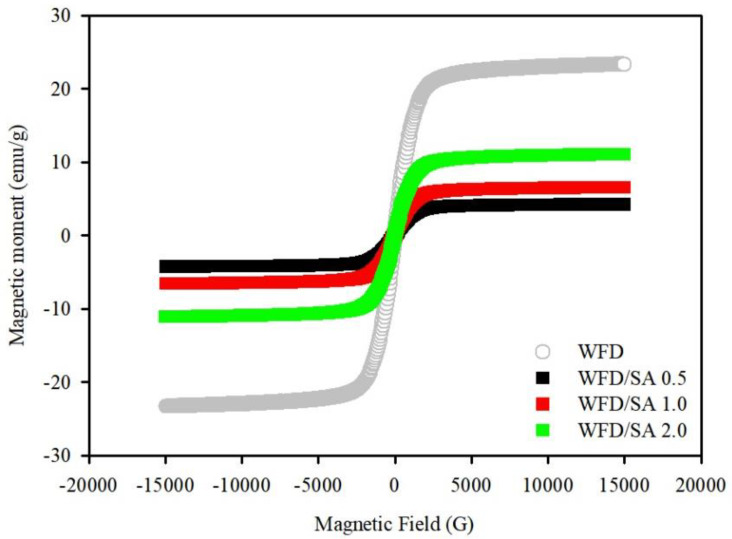
Magnetization curves of WFD and WFD/SA bead (different mass ratios of WFD).

**Figure 5:**
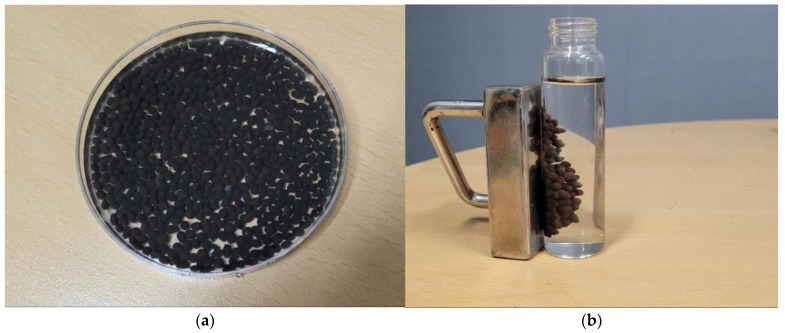
(**a**) A photo of WFD/SA-PEI and (**b**) photo to show magnetic properties of WFD/SA-PEI in solutions.

WFD/SA beads with different WFD mass ratios were tested. A comparison of different amounts of WFD showed that the magnetic saturation effect of the microspheres increased with increasing WFD content (Figure 4). The magnetic saturation of different amounts of WFD/SA bead was at 4.24 to 11.1 emu/g, which is lower than the magnetization saturation of WFD (23.3 emu/g), which was possibly due to the shielding of WFD by alginate. As expected, the recovery rate of the WFD/SA bead depended on the intensity of magnetism. Based on the comparison of the magnetic saturation, WFD/SA (2 wt%) was selected as the optimal adsorbent (Figure 5).

**Figure 6:**
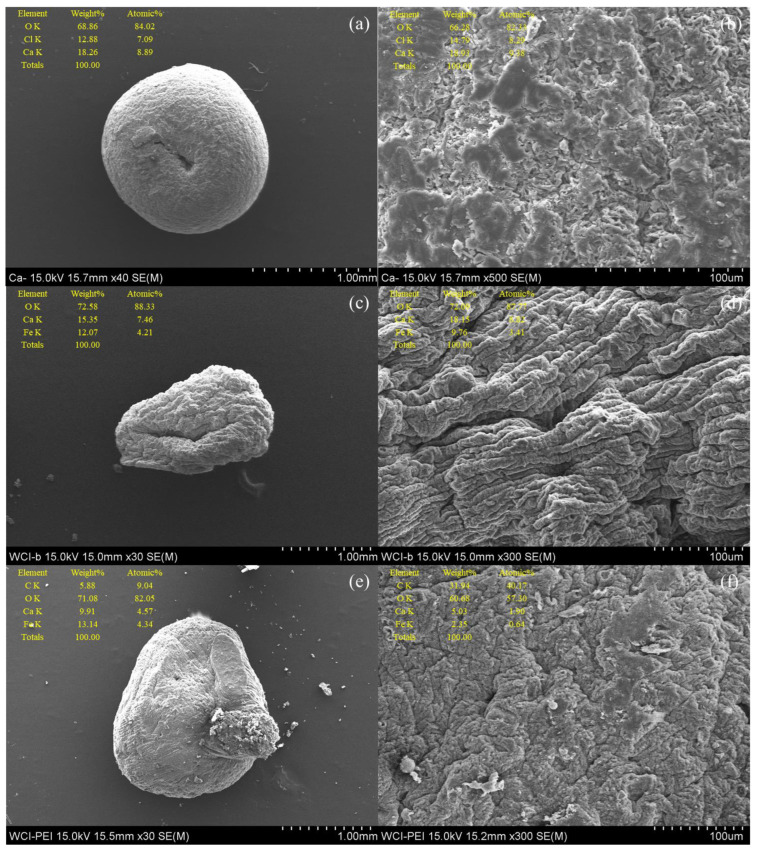
SEM images and EDS analysis of element content of (**a**,**b**) Ca-alginate bead, (**c**,**d**) WFD/SA bead, and (**e**,**f**) WFD/SA-PEI bead at two different magnifications.

The FE-SEM images and EDS analyses are shown in Figure 6. The morphologies of Ca-alginate, WFD/SA, and WFD/SA-PEI beads showed similar microstructures, indicating that the encapsulation of WFD particles in the Ca-alginate beads did not change the original structure. This may have resulted from encapsulation due to the interactions between WFD and SA, resulting in denser beads. Additionally, it was revealed that the pores were not observed clearly on the surface, and a relatively fragmentary structure caused shrinkages and dents on their surfaces after drying. EDS was then performed to analyze the elemental distribution on the surface of the beads. WFD/SA beads showed that Ca, O, and Fe were detected, which is primarily attributed to the SA crosslinking as the immobilization for Ca, due to the ionic gelation of alginate, in which the Na^+^ in the alginate structure is replaced by Ca^2+^ [35].

Additionally, from the N_2_ adsorption–desorption analysis, the BET specific surface area and total pore volume of WFD/SA-PEI were determined to be 1.06 m^2^/g and 0.002 cm^3^/g, respectively.

**Figure 7:**
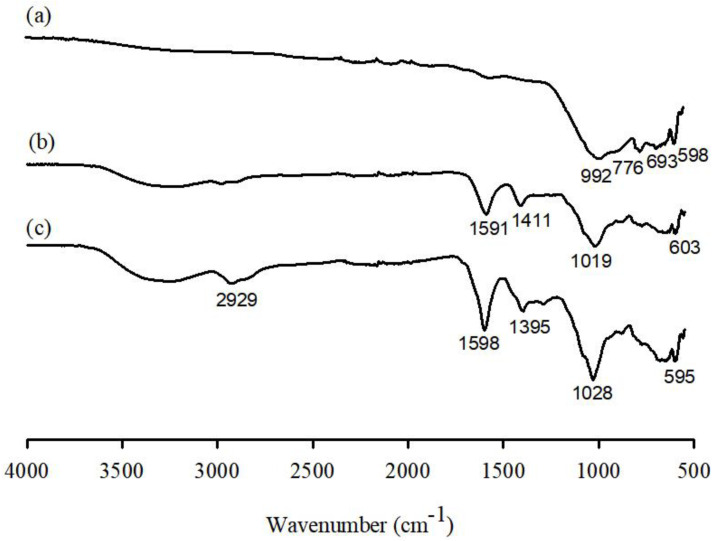
Fourier transform infrared spectroscopy measurements of WFD, WFD/SA bead and WFD/SA-PEI bead.

The FTIR spectra are shown in Figure 7. In the spectra of WFD (Figure 7(a)), the peaks at 992, 776, and 693 cm^−1^ were attributed to Si-O stretching vibration bands, whereas the peaks at 598 cm^−1^ corresponded to F-O bond stretching. Typically, the broad band present between 3400 and 3200 cm^−1^ in the spectra represents the stretching vibration of the O-H bonds of SA (Figure 7 (b). The peaks at 1591 and 1411 cm^−1^ correspond to the asymmetric and symmetric stretching vibrations of the C-O bond of the COO– group, respectively [36,37,38]. This indicates the involvement of the COO− group in the Ca^2+^-mediated processes of alginate reticulation and egg-box structure formation [34]. Moreover, the band at 1019 cm^−1^ is attributed to the C-O stretching [39]. Similar absorption bands have been reported previously [40,41]. After PEI was grafted onto the WFD/SA surfaces (Figure 7 (c), the characteristic broad band at 3200–3600 cm^−1^ was attributed to the stretching vibrations of O-H and N-H groups. A notable peak observed at 2929 cm^−1^ was considered to be due to C-H stretching vibration in –CH. The peak of the –COO- (asymmetric) stretching vibration shifted to 1598 and 1395 cm^−1^, which could be attributed to the hydrogen-bonding effect of the N-H groups in PEI [42,43,44]. This could be attributed to the –NH_2_ and OOC- bonds formed by electrostatic attraction between the oppositely charged PEI and alginate [45].

**Figure 8:**
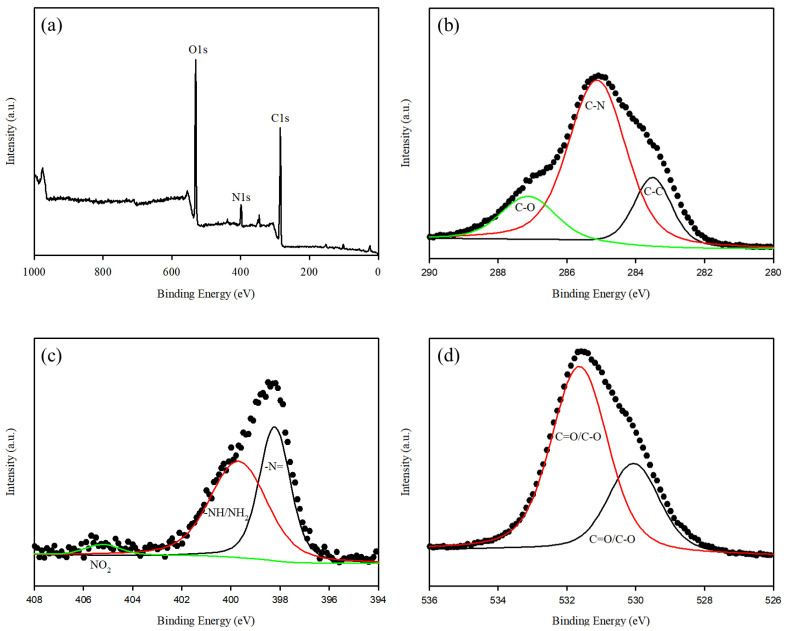
XPS spectra of WFD/SA-PEI bead: (**a**) wide scan and high-resolution XPS spectra of (**b**) C 1s, (**c**) N 1s and (**d**) O 1s.

The XPS spectrum of the WFD/SA-PEI beads is presented in Figure 8, where the main elements (C and N) were recorded in the ranges of 280–290 and 394–406 eV, respectively. The photoelectron peak at binding energies of 284.9 was attributed to C1s orbitals and the peaks at 399.0 and 531.3 eV were assigned to N1s, and O1s orbitals, respectively [46]. In a high-resolution scan of the C 1s section, the peaks at 283.4, 285.0 and 287.0 eV were attributed to C-C, C-N, and C-O bonds, respectively [47]. The oxygen (O1s) spectrum was deconvoluted into peaks at 530.0 and 531.6 eV, respectively, which are consistent with the characteristic binding energies of the C=O/C-O groups. In the N1s spectrum, three deconvoluted peaks were assigned to N in –N= (398.2 eV), –NH/–NH_2_ (399.6 eV) and NO_2_ (405.2 eV) [48,49]. This confirms the successful modification of the WFD/SA matrix using PEI.

### 3.3. MR Removal under Batch Conditions

#### 3.3.1. Effect of Initial pH on the Adsorption

**Figure 9:**
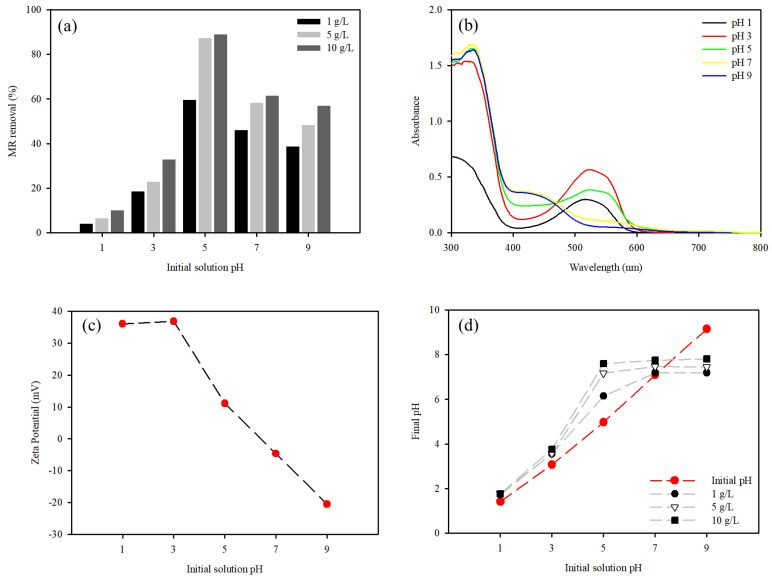
(**a**) MR adsorption to WFD/SA-PEI bead at solution pH ranging from 1 to 9. (**b**) UV-Vis spectrum of MR at various pH values. (**c**) Zeta potential of the WFD/SA-PEI bead as a function of solution pH. (**d**) Effect of the initial pH on the final pH of the solution reacted with the WFD/SA-PEI bead.

The pH of the solution is generally considered an important parameter in the adsorption processes and removal capacity. The effect of solution pH on dye removal was examined in the pH range of 1–9. The MR removal capacity was highest at pH 5 with a removal capacity in the range of 1.0–10 g/L of 672.7 mg/g (59.7%), 185.4 mg/g (87.3%) and 94.2 mg/g (89.1%), respectively. As the dose of the WFD/SA-PEI beads in solution increased, the number of sorption sites available for MR increased, which then provided more functional groups, resulting in increased MR removal. However, the MR adsorption capacity decreased with increasing doses of WFD/SA-PEI beads (Figure 9a).

As the solution pH decreased toward highly acidic conditions, the MR removal capacity decreased. For instance, the MR removal capacity was 218.1 mg/g at pH 3. As the pH decreased to 1, the removal capacity sharply increased to 36.6 mg/g. This can be attributed to the different behaviors of MR in aqueous solutions. This trend is explained by the fact that ${\mathrm{HMR}}^{+}$ and ${\mathrm{MR}}^{-}$ represent the protonated (acid) and deprotonated (basic) forms of MR (Equation (9)).

(9) $${\mathrm{HMR}}^{+}\left(\mathrm{acid}\right)\overset{H^{+}}{\Leftrightarrow }\mathrm{MR}\overset{H^{+}}{\Leftrightarrow }{\mathrm{MR}}^{-}\left(\mathrm{basic}\right)$$

The color of the MR solution varies significantly with the variation in the initial pH. The characteristic peaks of the initial solution pH of the MR are shown in Figure 9b. The spectra revealed differences in their patterns. It can be seen that the absorption maximum wavelength in the visible range is λ = 420 and 520 nm in basic and acidic solutions, respectively. This is because the color of the MR changes upon protonation or deprotonation [50,51]. During MR adsorption, the adsorption of charged MR groups is influenced by the surface charge of the adsorbent. The zeta potential results showed that the WFD/SA-PEI beads had an isoelectric point around pH 6.3, which was mainly due to the introduction of a large number of amino groups on the surface (Figure 9c). The above results indicated that when pH < 6.3, the WFD/SA-PEI bead surface charge was positive, and when pH > 6.3, the surface charge was negative. When pH < 6.3, the amine groups on PEI were well protonated into ammonium groups (Equation (10)).

(10) $$-{\mathrm{NH}}_{2}+H^{+}↔-{\mathrm{NH}}_{3}^{+}$$

Therefore, under highly acidic pH conditions, electrostatic repulsion between the positively charged adsorbent surfaces and positively charged MR ions can occur. In contrast, the amine groups on PEI can be partially converted to deprotonated amine groups with increasing pH (Equation (11)).

(11) $$-{\mathrm{NH}}_{2}+{\mathrm{OH}}^{-}↔-{\mathrm{NH}}_{2}{\mathrm{OH}}^{-}$$

With a further increase in pH (>6.3), the adsorption capacity decreased, but the WFD/SA-PEI beads still had a high adsorption capacity. All the final pH values of the solution increased from the initial pH values, with the exception of the initial pH of 9 (Figure 9d). This indicates that the pH remained almost constant, and the slight increase in pH was possibly due to the MR^−^ exchange with OH^−^. Meanwhile, the initial pH of 9 for the adsorption process decreased to pH 7.19–7.80. This is because as the pH increases, the overall zeta potential decreases and the negatively charged MR increases, whereas the positive charge on the surface decreases as the amine groups become deprotonated.

#### 3.3.2. Kinetic and Equilibrium Model Analyses

**Figure 10:**
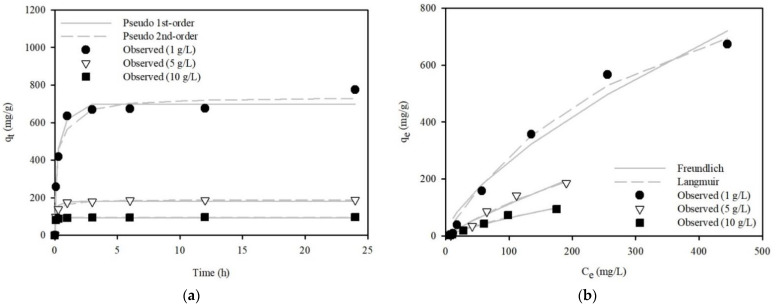
(**a**) MR adsorption kinetics fitted by pseudo-first-order and pseudo-second-order models onto the WFD/SA-PEI bead. (**b**) Adsorption isotherm of MR onto the WFD/SA-PEI bead (pH: 4.0; contact time: 3 h).

MR removal by WFD/SA-PEI beads was evaluated at initial concentrations (C_0_ = 1000 mg/L) over a 24 h period with different amounts of WFD/SA-PEI beads. The MR removal capacity of the WFD/SA-PEI beads reached equilibrium in 3 h. To investigate the mechanism of MR removal by the WFD/SA-PEI beads and determine the rate-controlling factors, batch study data were analyzed using kinetic sorption models, such as pseudo-first-order and pseudo-second-order models. The kinetic adsorption data and model analysis are shown in Figure 10a. Equations (2)–(7) were applied to the experimental data for the adsorption of the anionic dye MR onto the WFD/SA-PEI beads. The kinetic model parameters are listed in Table 4. The pseudo-second-order model best fitted the kinetic data. The MR sorption data as a function of the initial MR concentration in the WFD/SA-PEI beads were analyzed using equilibrium isotherm models, including the Langmuir and Freundlich models. The effect of the initial MR concentration on adsorption is presented in Figure 10b. The equilibrium model parameters are listed in Table 5. The R^2^, χ^2^, and SSE values indicate that the Langmuir model is the most suitable for describing the equilibrium data. The MR removal capacities of various low-cost adsorbents reported in the literature are presented in Table 6.

#### 3.3.3. Effect of Coexisting Ions for the Removal of MR

**Figure 11:**
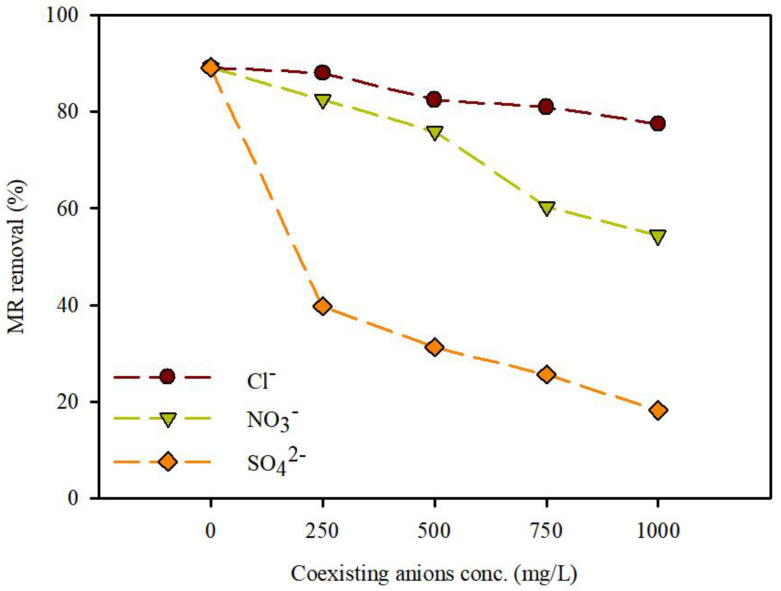
Effects of coexisting ions on MR adsorption by WFD/SA-PEI bead (initial concentration: 1000 mg/L; pH: 4.0).

In textile wastewater, there are often various inorganic anions and organic matter that affect the adsorption capacity. Therefore, the effects of coexisting ions, i.e., Cl^−^, NO_3_^−^, and SO_4_^2−^, on the adsorption of MR by WFD/SA-PEI beads were investigated. The concentration of MR was maintained at 1000 mg/L, while the co-existing ion concentration increased from 200 to 1000 mg/L with a contact time of 180 min. Our results showed that the adsorption of MR in the presence of coexisting ions was affected (Figure 11). The MR removal capacities of the WFD/SA-PEI beads decreased with increasing concentrations of Cl^−^, NO_3_^−^, and SO_4_^2−^. The relative removal rate decreased from 88.0% to 77.4% as the Cl^−^ concentration increased from 200 mg/L to 1000 mg/L. Within the same range, the relative removal rate of SO_4_^2−^ decreased from 39.7% to 18.2%. The influence of coexisting anions on MR removal followed the order Cl^−^ > NO_3_^−^ > SO_4_^2−^. This result could be attributed to the fact that the ionic radius of multivalent anions is larger than that of monovalent anions, which could be ascribed to the competition between the MR molecules and coexisting anions for sorption sites [35]. In other words, Cl^−^ and NO_3_^−^ are monovalent anions, which slightly compete with MR molecules for the positively charged active sites on the WFD/SA-PEI beads. However, SO_4_^2−^ is a multivalent anion that can compete with the MR for active sites, resulting in a weakened adsorption capacity. This result indicated that the WFD/SA-PEI beads exhibited selectivity for MR adsorption from wastewater containing coexisting ions.

### 3.4. MR Removal Mechanisms of WFD/SA-PEI Bead

The protonation status of MR is pH-dependent, which results in a change in color and aqueous solubility. MR has been reported to have a dissociation constant (pKa) of 5.1, and contains –COOH and N–H groups [15]. In other words, MR molecules are positively charged at pH < pKa and negatively charged at pH > pKa. This result could be attributed to the MR being easily the self-auto-ionization of water, which released H^+^ [34]. This phenomenon can be explained by the shift in the absorption maximum wavelength from 420 to 520 nm.

A simple method of forming a positively charged WFD/SA-PEI bead was developed using PEI on the WFD/SA surface, involving the linking of PEI amino groups to the SA surface carboxyl groups. Batch experiments revealed that the adsorption process occurs mainly via electrostatic forces. The surface charge of the WFD/SA-PEI beads was positive when the solution pH was <pH_PZC_. However, the surface charge was negative when the pH of the solution was greater than that of pH_PZC_. This indicates that the WFD/SA-PEI beads are related to the pH of the solution and have a proton-buffering capacity by amino groups present in PEI [60]. Our results indicate that MR adsorption at low pH was far lower than that under neutral and weakly basic solution conditions. This could be attributed to the restriction of MR removal to the sorption sites by the large number of hydrogen ions present in the aqueous phase at a highly acidic pH. In other words, an increase in the H^+^ ions leads to competition with positively charged MR. However, the MR removal capacity was at its highest at pH 5. The enhanced removal efficiency may be due to the presence of PEI, which offers more binding sites (–NH_2_ and OH groups) for the uptake of MR molecules. In addition, the MR molecules have free –COOH and N–H groups, which can form hydrogen bonds with the –NH_2_ and OH groups present in the WFD/SA-PEI beads. However, the positive charge on the surface decreases with increasing pH, which is partially caused by the deprotonated -NH_2_ groups.

## 4. Conclusions

Easily synthesized and recyclable materials were prepared for MR removal from aqueous solution. The WFD was used as a cost-effective material for magnetic Fe_3_O_4_, providing a possible path for recycling. A magnetic adsorbent was developed by encapsulating WFD in calcium-alginate beads. The WFD/SA beads were then functionalized using PEI. FTIR and XPS analyses proved that PEI was successfully crosslinked with WFD/SA beads. It was found that the presence of the functional groups in SA and PEI on the surface aided in the adsorption of MR. The adsorption of MR by these WFD/SA-PEI beads was studied and the effects of different parameters, such as solution pH, initial concentration, contact time, and coexisting ions on the adsorption of MR, were investigated. The variation in pH brings about structural changes through the protonation or deprotonation of the MR molecules based on the pKa value, and the MR adsorption by WFD/SA-PEI beads also depends on the functional groups present on the adsorbent as the pH is varied. The removal mechanism of MR on the WFD/SA-PEI beads was mainly achieved via electrostatic adsorption and hydrogen bonding. Accordingly, the WFD/SA-PEI beads proved to be an economical and efficient adsorbent for the removal of anionic dyes from wastewater.

## Figures and Tables

**Table 1:** Properties of MR used in this study.

Dye	Color	pKa	Change Range of pH	Molecular Weight (g/mol)	Maximum Adsorption Wavelength (nm)	Chemical Structure
Methyl red	Red	5.1	4.4 < pH < 6.0	269.3	520	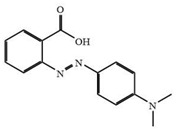

**Table 2:** Chemical composition of the WFD.

**Compound**	**Fe_2_O_3_**	**SiO_2_**	**ZrO_2_**	**Al_2_O_3_**	**SO_3_**
Conc. (%)	40.03	25.45	10.09	7.28	4.03
**Compound**	**CuO**	**ZnO**	**PdO**	**K_2_O**	**CaO**
Conc. (%)	2.45	1.99	1.87	1.48	1.44

**Table 3:** Leaching results of heavy metals elements from the WFD at various pH values by the modified KSLT (unit: mg/L, N.D: Not detected).

**Sample**	**Final pH**	**Al**	**As**	**Ba**	**Ca**	**Cd**	**Co**	**Cr**	**Cu**
pH1	1.53	143.36	0.58	0.13	503.10	N.D	N.D	0.08	205.94
pH3	5.24	11.52	N.D	N.D	258.98	N.D	N.D	N.D	1.90
pH5	5.58	6.55	N.D	N.D	202.73	N.D	N.D	N.D	1.14
pH7	5.67	6.23	N.D	N.D	199.75	N.D	N.D	N.D	0.88
pH9	5.68	6.50	N.D	N.D	217.35	N.D	N.D	N.D	1.05
**Sample**	**Fe**	**K**	**Mg**	**Mn**	**Mo**	**Na**	**Ni**	**Pb**	**V**
pH1	156.51	74.58	38.41	95.00	0.34	94.13	N.D	15.77	0.97
pH3	N.D	38.13	27.58	32.48	0.06	80.48	N.D	N.D	N.D
pH5	N.D	23.66	15.78	17.70	N.D	47.93	N.D	N.D	N.D
pH7	N.D	23.45	14.81	17.21	N.D	45.79	N.D	N.D	N.D
pH9	N.D	28.07	20.32	23.01	N.D	59.79	N.D	N.D	N.D

**Table 4:** Kinetic model parameters obtained from model fitting to experimental data.

**Adsorbent Dose** **(g/L)**	**Pseudo-First-Order Model**				
	***q_e_*** **(mg/g)**	***k*_1_** **(1/h)**	***R* ^2^**	** $\chi^{2}$ **	**SSE**
1	698.41	2.15	0.97	2.64 × 10^1^	1.23 × 10^4^
5	180.97	4.14	0.97	4.32 × 10^0^	6.46 × 10^2^
10	93.14	11.77	0.99	5.93 × 10^−1^	5.51 × 10^1^
**Adsorbent Dose** **(g/L)**	**Pseudo-Second-Order Model**				
	***q_e_*** **(mg/g)**	***k*_2_** **(g/mg/h)**	***R* ^2^**	** $\chi^{2}$ **	**SSE**
1	737.79	0.004	0.97	1.90 × 10^1^	1.14 × 10^4^
5	190.11	0.03	0.99	9.38 × 10^−1^	1.47 × 10^2^
10	95.05	1.09	0.98	1.56 × 10^0^	1.42 × 10^2^

**Table 5:** Equilibrium isotherm model parameters obtained from model fitting to experimental data.

**Adsorbent Dose** **(g/L)**	**Freundlich Model**				
	***K_F_*** **(L/g)**	**1/*n***	***R* ^2^**	** $\chi^{2}$ **	**SSE**
1	11.83	0.67	0.96	8.78 × 10^1^	1.351 × 10^4^
5	1.94	0.87	0.96	2.04 × 10^1^	9.924 × 10^2^
10	1.45	0.81	0.97	1.00 × 10^1^	2.04 × 10^2^
**Adsorbent Dose** **(g/L)**	**Langmuir Model**				
	***Q_m_*** **(mg/g)**	***K_L_*** **(L/mg)**	***R* ^2^**	** $\chi^{2}$ **	**SSE**
1	1203.91	0.003	0.99	4.00 × 10^1^	3.93 × 10^3^
5	704.10	0.001	0.97	1.74 × 10^1^	7.66 × 10^2^
10	262.15	0.003	0.98	7.14 × 10^0^	1.13 × 10^2^

**Table 6:** MR removal capacities of various low-cost adsorbents reported in the literature.

Adsorbent	Removal Capacity (mg/g)	Reference
BPEI-modified magnetic activated carbon	526	[52]
Lemongrass leaf-based activated carbon	72.3	[53]
Fe_3_O_4_@MIL-100(Fe)	686.3	[54]
Thiosemicarbazide-modified chitosan (TSFCS)	17.3	[55]
Natural and Purified Organic Matter Rich Clays	397	[56]
N, N-Dimethyldodecylamine N-oxide(DDAO)–coffee residues(CR)	76.7	[57]
Sewage sludge blended with waste coal	312.7	[58]
Fe_3_O_4_@SiO_2_@NH_2_, amorphous silica from rice husk	81.3	[59]
WFD/SA-PEI	672.7	This study

## Data Availability

All data generated or analyzed during this study are included in this published article.
